# Exercise Regulation of Marrow Adipose Tissue

**DOI:** 10.3389/fendo.2016.00094

**Published:** 2016-07-14

**Authors:** Gabriel M. Pagnotti, Maya Styner

**Affiliations:** ^1^Department of Biomedical Engineering, Stony Brook University, Stony Brook, NY, USA; ^2^Department of Medicine, University of North Carolina, Chapel Hill, NC, USA

**Keywords:** exercise, marrow adipose tissue, quantitative image analysis, bone microarchitecture, lipid, PPARγ, rosiglitazone, exercise

## Abstract

Despite association with low bone density and skeletal fractures, marrow adipose tissue (MAT) remains poorly understood. The marrow adipocyte originates from the mesenchymal stem cell (MSC) pool that also gives rise to osteoblasts, chondrocytes, and myocytes, among other cell types. To date, the presence of MAT has been attributed to preferential biasing of MSC into the adipocyte rather than osteoblast lineage, thus negatively impacting bone formation. Here, we focus on understanding the physiology of MAT in the setting of exercise, dietary interventions, and pharmacologic agents that alter fat metabolism. The beneficial effect of exercise on musculoskeletal strength is known: exercise induces bone formation, encourages growth of skeletally supportive tissues, inhibits bone resorption, and alters skeletal architecture through direct and indirect effects on a multiplicity of cells involved in skeletal adaptation. MAT is less well studied due to the lack of reproducible quantification techniques. In recent work, osmium-based 3D quantification shows a robust response of MAT to both dietary and exercise intervention in that MAT is elevated in response to high-fat diet and can be suppressed following daily exercise. Exercise-induced bone formation correlates with suppression of MAT, such that exercise effects might be due to either calorie expenditure from this depot or from mechanical biasing of MSC lineage away from fat and toward bone, or a combination thereof. Following treatment with the anti-diabetes drug rosiglitazone – a PPARγ-agonist known to increase MAT and fracture risk – mice demonstrate a fivefold higher femur MAT volume compared to the controls. In addition to preventing MAT accumulation in control mice, exercise intervention significantly lowers MAT accumulation in rosiglitazone-treated mice. Importantly, exercise induction of trabecular bone volume is unhindered by rosiglitazone. Thus, despite rosiglitazone augmentation of MAT, exercise significantly suppresses MAT volume and induces bone formation. That exercise can both suppress MAT volume and increase bone quantity, notwithstanding the skeletal harm induced by rosiglitazone, underscores exercise as a powerful regulator of bone remodeling, encouraging marrow stem cells toward the osteogenic lineage to fulfill an adaptive need for bone formation. Thus, exercise represents an effective strategy to mitigate the deleterious effects of overeating and iatrogenic etiologies on bone and fat.

## Marrow Adipose Tissue

Increased marrow adipose tissue (MAT) is associated with states of impaired bone formation (1, 2) and dysfunctional hematopoiesis (3–5), although its physiological role remains unclear. In humans, pathologists have noted that MAT increases in healthy subjects with age, beginning in the distal long bones and accruing proximally such that by age 25, approximately 70% of the marrow space is filled with MAT (4). In addition to physiologic MAT, which accrues with aging, this fat depot – housed within bone – is abundant in states of low bone density: osteoporosis (6), anorexia nervosa (7), skeletal unloading (8, 9), and anti-diabetes therapies (10), conditions that are also associated with skeletal fractures. Adipocytes within the marrow originate from the mesenchymal stem cell (MSC) pool that also gives rise to osteoblasts, chondrocytes, and myocytes, among other cell types (11, 12). Recent work suggests that increased marrow fat can also be demonstrated in the setting of preserved or increased bone density (high-fat feeding or obesity) (13–15) and, thus, challenges the premise that the relationship between MAT and bone volume is reciprocal. The MAT/bone relationship is further complicated by the identification of a new population of Grem1^+^ MSC (16), a phenotype capable of differentiating into osteoblasts and chondrocytes, but not adipocytes: the Grem1^+^ population differs from the LepR^+^ MSC, which do generate marrow adipocytes. Whether senile marrow invasion with adipocytes represents a later predominance of a LepR^+^ MSC population is unknown but complicates considerations as to the physiologic and/or pathologic role of MAT.

In the case of diet-induced obesity, marrow fat also increases compared to normal weight controls, but whether this contributes to bone fragility is unclear (17). Nevertheless, if the burden of fat across the marrow space is inevitable, then perhaps what’s more worthy of an investigation is the quality of the MAT being generated, possibly representing a direct reflection of the health of the surrounding bone. Importantly, the unsaturation index of MAT increases with aging, and thus, this feature of MAT may shed light on its physiology; nonetheless, unsaturation index of MAT is unaffected by physical activity (18). While subcutaneous white fat depots store excess energy and provide a clear evolutionary advantage during times of scarcity (19), MAT’s purpose remains indeterminate, harboring characteristics of both white and brown fat (20). WAT serves as a source of adipokines and inflammatory markers that have both positive (e.g., adiponectin) (21) and negative (22) effects on metabolic and cardiovascular endpoints. Visceral abdominal fat is a distinct depot of WAT that is proportionally associated with negative metabolic and cardiovascular morbidity (23), regenerates cortisol (24), and has been linked to reduced bone formation (25, 26). WAT substantially differs from brown adipose tissue (BAT), as defined by a panel of proteins that support BAT’s thermogenic role (27). MAT, by virtue of its specific marrow location and its adipocyte origin from at least LepR^+^ marrow MSC, is clearly demarcated from non-bone fat depots by higher expression of bone transcription factors (28) and likely represents a unique fat phenotype (29). Recently, MAT was noted to produce a greater proportion of adiponectin – an adipokine associated with improved metabolism – than WAT (30), suggesting an endocrine function for MAT as distinct depot, akin, but different from that of WAT. Moreover, deficiency of histone deacetylase 3 (Hdac3), known to play a major role in skeletal development and lipid metabolism, increases MAT volume, implicating this important transcriptional regulator in MAT development (31). Potentially, MAT might serve multiple functions, reflecting those of both white and brown fat, storing lipid in preexisting adipocytes, secreting adipokines, and generating heat. Exercise universally affects the metabolism of both WAT (32) and more recently BAT (33, 34); thus, exercise intervention can be harnessed as a powerful tool to query the poorly understood physiology of MAT.

## Measurement and Quantification of Marrow Adipose Tissue

In order to quantify and characterize the effects of exercise on MAT, various analytic methods were considered. Until recently, qualitative measurements of MAT have relied on bone histology (35, 36), which is subject to site selection bias and cannot adequately quantify the volume of fat in the marrow. Nevertheless, histological techniques and fixation make possible *in situ* visualization of MAT, quantification of adipocyte size, and MAT’s association with the surrounding endosteum, milieu of cells, and secreted factors (37–39).

Recent advances in cell surface and intracellular marker identification and single-cell microfluidic analyses have led to greater resolution and high-throughput *ex vivo* quantification. Flow cytometric quantification can be used to purify adipocytes from the stromal vascular fraction of most fat depots (40). Early research with such machinery cited adipocytes as too large (50–200 mm) and fragile for cytometer-based purification, as their cytoskeleton lacks rigidity, rendering them susceptible to lysis; however, recent advances have been made to mitigate this (41). One may distinguish discrete adipocyte subpopulations from other cells by utilizing internal lipid content and surface biomarker identification. Filtration of the marrow (pore size = 150 μm) permits adequate flowthrough for adipocytes and smaller cellular contents. Subsequent centrifugation of the suspension aids in isolating adipocytes. Maintaining laminar flow and optimal temperatures when sorting has led to greater viability and precision. However, accumulation of lipid and protein content can adhere to the sheath tubing, thereby clogging the instrumentation (42). High-binding affinity of protein to antibodies bound by fluorescent probes used in FACS has made the identification of MAT and the cells that cohabitate the marrow increasingly specific, though these measurements provide little information on adipocyte location within the marrow microenvironment.

**Figure 1:**
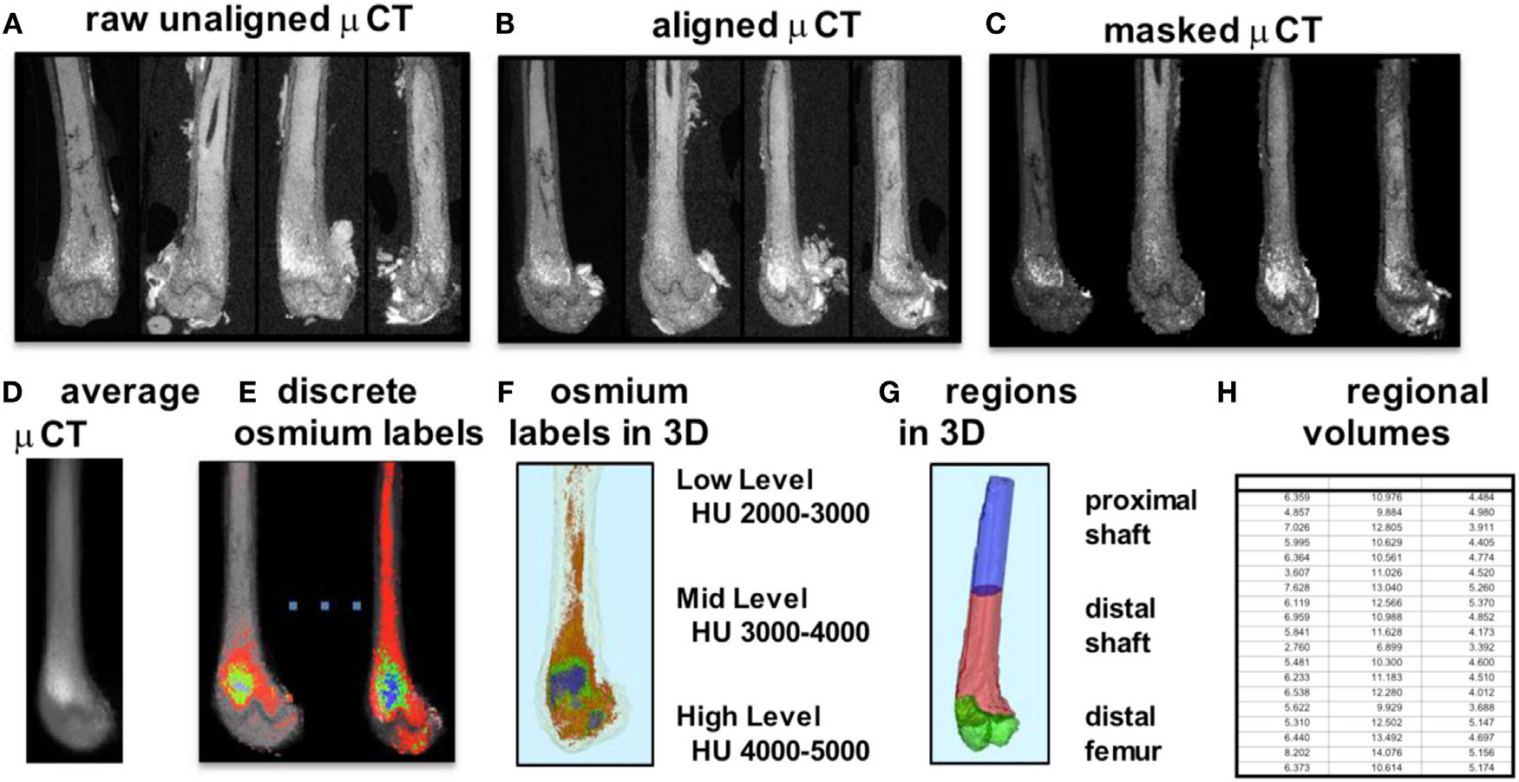
**Overview of method for visualization and quantification of marrow adipose tissue (MAT)**. Osmium-stained femorae are visualized *via* μCT. Femorae **(A)** are rigidly aligned **(B)**. Bone masks **(C)** are averaged **(D)**. Osmium within the bone mask is quantified as volumetric (cubic millimeter) measurements of low (red), mid (green), and high (blue) osmium-containing regions in the femur and **(E)** overlaid on μCT images for viewing. 3D rendering of osmium regions **(F)** with same coloring as **(E)**, colors slightly offset due to transparent bone mask. In **(G)**, the femur is subdivided into three anatomical regions of interest. **(H)** is a pictorial representation of a data spreadsheet containing regional osmium measurements as osmium volume normalized to bone volume (in %).

**Figure 2:**
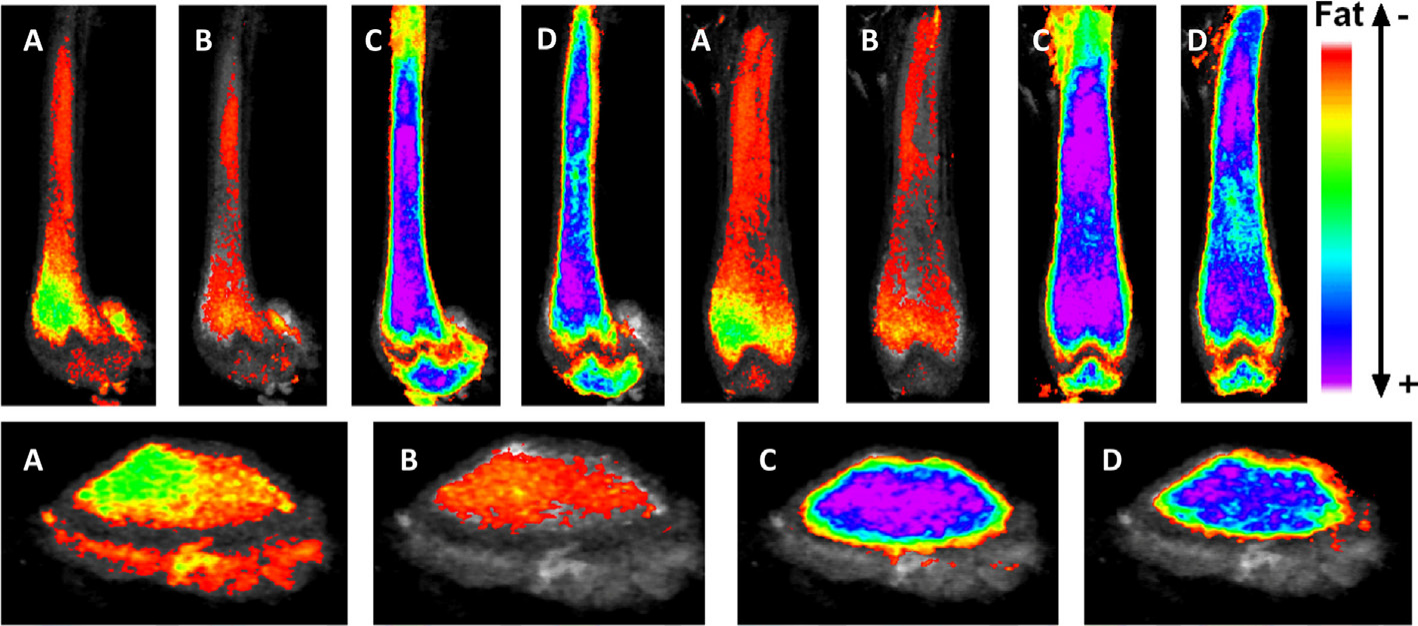
**Exercise suppresses marrow adipose tissue accumulation, despite PPARγ agonist treatment**. Visualization of osmium (lipid-binder) stain by μCT in sagittal (top left), coronal (top right), and axial (bottom) planes in the femur of C57BL/6 mice. Visualization is performed by superimposing and averaging the images of each femur (*n* = 5 per group) and colored labeling of osmium according to Hounsfield unit (HU) density. The four experimentals are as follows: control **(A)**, rosiglitazone **(B)**, control-exercise **(C)**, and rosiglitazone-exercise **(D)**.

To improve our understanding of MAT, novel imaging techniques have recently been developed as a means to visualize and quantify MAT, *in situ*. Although proton magnetic resonance spectroscopy (1H-MRS) has been used with success to quantify vertebral MAT in humans (43), it is more difficult to employ in laboratory animals (44). Magnetic resonance imaging (MRI) provides MAT assessment in the vertebral skeleton (45) in conjunction with μCT-based marrow density measurements (46). A volumetric method to identify, quantify, and localize MAT in rodent bone has been recently developed, requiring osmium staining of bones and μCT imaging (47), followed by image analysis of osmium-bound lipid volume (in cubic millimeter) relative to bone volume (see Figure 1) (13, 48). Briefly, femurs stripped of connective tissue are decalcified, immersed in osmium tetroxide, and placed in potassium dichromate (49). Bones are scanned using μCT imaging (resolution 10 μm × 10 μm × 10 μm). Image processing consists of rigid image coalignment (Figures 1B,D, Slicer) (50), regional masking, allowing consistent regional measurements and superimposed visualizations. Additional bone masks are established in a semiautomatic contouring of the femur. As osmium is significantly more dense than bone [Hounsfield units (HU) ~ 700–2000], HU thresholds are set to capture low osmium from 2000 to 3000 HU, mid osmium from 3000 to 4000 HU (Figures 1E,F, green), and high osmium from 4000 to 5000 HU (Figures 1E,F, blue), and quantified accordingly (cubic millimeter). The lowest threshold is set above dense cortical bone (2000 HU) (51), and, thus, the contribution of potentially mislabeled cortical bone to the osmium volume is negligible (51, 52). Following quantification, the femur is subdivided into anatomical regions wherein regional osmium volume is normalized to bone volume. Aligned bone images are then averaged across all images as a reference for visualization. Average images are also computed for each group to obtain color-coded visualizations of the osmium densities to allow additional visual comparison of MAT between groups (Figure 2). This technique provides reproducible quantification and visualization of MAT, enabling the ability to quantify changes in MAT with diet, exercise, and agents that constrain precursor lineage allocation.

## Exercise Regulation of Marrow Adipose Tissue in the Setting of High-Fat Feeding

Marrow adipose tissue volume has recently been demonstrated to increase during short-term, high-fat feeding in rodents relative to the total bone volume (13), rising more rapidly than calorie induction of visceral fat depot size (53). A positive association between obesity and MAT was noted in rodents (15) and humans (43). In fact, obese individuals generally present with higher bone density (54–62) and are unlikely to experience classical osteoporotic fragility fractures (63). Because MAT has been associated with states of low bone density and increased fracture risk (6, 8, 64), the impact of fat in the marrow likely varies depending on its etiology. As obesity associates with increased bone density and lower fracture, MAT in obesity could represent a distinct fat depot that supports skeletal anabolism.

The *Nurse’s Health Study* showed that exercise affords a morbidity and mortality benefit that is independent of weight loss or calorie expenditure (65). One well-known effect of exercise is to improve bone strength, limiting the impact of postmenopausal osteoporosis (66). Exploring the salutary effects of exercise on bone and MAT in the setting of HFD, small animal models have been employed to address the degree of responsivity under said conditions. In one such experiment, 6 weeks of daily voluntary running exercise suppresses MAT accumulation in mice fed both a regular and HFD (13), in contrast to their non-exercised counterparts. While HFD does not perturb trabecular bone parameters as compared to control, significant gains in trabecular bone volume and trabecular thickness are noted in tibiae of exercised animals. Cortical bone parameters are unaltered by HFD and exercise in this short-term study. Thus, the trabecular compartment might be more receptive to both metabolic disarray and to mechanical signals from exercise than cortical bone, especially when challenged with elevated MAT *via* HFD.

## Exercise Regulation of Marrow Adipose Tissue in the Setting of Rosiglitazone

It is accepted that elevated MAT due to PPARγ-agonist treatment in mice (48, 49, 67) and humans (68, 69) is due to PPARγ-induction of adipogenesis from marrow MSC. *In vitro*, rosiglitazone (PPARγ-agonist)-treated MSCs resist mechanically stimulated adipogenesis when treated with a dynamic load (70). *In vivo*, it was previously hypothesized that exercise might not overcome pathologically induced MAT in the setting of rosiglitazone; however, interestingly, exercise suppresses MAT even in the face of powerful adipogenic biasing (*via* MSC) in rosiglitazone-treated mice (48). While rosiglitazone significantly elevates cortical porosity in proximal tibiae, bone quantity is unaffected in these young mice. Exercise associates with increased trabecular bone volume fraction and trabecular thickness in control and rosiglitazone-treated mice (48). This further highlights the positive impact of exercise and mechanical signals on healthy bone as well as bone challenged by pharmacologic agents shown to facilitate marrow adiposity as well as cortical porosities.

## Exercise and Mechanical Regulation of Bone Quality and Quantity

### Exercise and Musculoskeletal Mass

Of the many health benefits attributed to exercise – improved neurological endpoints (71, 72), cardiovascular health (73), reduced inflammation (74–77), and decreased risk for chronic disease development, perhaps, most immediately and visually apparent is its ability to augment musculoskeletal mass (78–81). Exercise is known to encourage anabolic responses in musculoskeletal tissue (i.e., bone, muscle, ligament, and tendon) (82) as a consequence of successive bouts of musculoskeletal-loading. Indeed, skeletal tissue is known to adapt to meet loading demands (83), altering its bone remodeling strategy to sustain maximum loads. Prominent and varied forces are exerted across the appendicular and axial skeleton (84) through exercise: the musculoskeletal construct, thereby, acts as a conduit for transducing muscle contractive perturbations at the bone surface at both low frequencies (e.g., bicep flexion) and high frequencies (type II fast-twitch muscle). At the cellular level, these responses are mediated by a wide spectrum of mechanical stimuli of both high- and low-magnitude stresses, information that is internalized by cells through cytoskeletal and transmembrane-bound integrins linking the extracellular environment with the genetic machinery encased within the nucleus (85–90). Thus, these mechanical factors transcribe osteo-, chondro-, or myogenic (91) growth factors (RunX2) while deterring pathways conducive to adipogenesis (PPARγ) (70, 92–95). We now know that these signals propagate across the Wnt/β–Catenin transduction pathways (96), upregulating expression of genes that drive osteogenic (RunX2) and chondrogenic (SOX9) growth, while also being positioned across other complex signaling pathways involving other signals of interest, such as MAPK, pRB, FGFs, and TGF-β. The ability of mechanical stimuli to regulate musculoskeletal mass is likely multifactorial, occurring through repression of fat generation as well as bone resorptive pathways (97), while at the same time stimulating musculoskeletal anabolism. Additionally, exercise has been shown to improve resistance to fracture, specifically through the separation of exercise into shorter regimens (98).

### Skeletal Unloading

The importance of mechanical information on bone adaptation can also be exemplified by observing the response of musculoskeletal tissue and the underlying cellular dynamics in the absence of mechanical cues (99–101). Whereas exercise delivers large quanta of mechanical information, as a function of disuse (chronic bed rest, microgravity, or reduced physical activity) (102, 103), regulation of musculoskeletal tissue homeostasis is compromised, instead elevating conditions wherein muscle, tendon, and ligament (and fat) undergo catalysis and rapid resorption of bone. Together, these tissue-level responses heighten the occurrence of osteoporotic bone and degree of fracture risk: these outcomes are in direct response to lapses in mechanical input (104–107). Whether chronic skeletal unloading encourages a specific MAT phenotype remains unclear, yet, studies have definitively shown that extended bed rest drives an increased marrow adipogenesis (108). In the absence of mechanical cues, PPARγ and receptor activator of nuclear factor-Kappa-B ligand (RANKL), which promotes osteoclast-mediated bone resorption, are both elevated, indicating an effect that could be stemmed upon reintroduction of mechanical stimuli.

### Mechanical Effect on Bone and Fat Precursors

Both *in vitro* and *in vivo* studies have demonstrated that MSCs and early, non-committed progenitors exhibit unquestionable responsivity to mechanical loading (93, 109). Osteocytes (88, 110), osteoblasts (111, 112), and pre-osteoblast MC3T3 cells (113) in the marrow are other known mechanosensitive cells and contribute to the complex transduction of mechanical information driving osteogenic gene expression. While mechanical signals can inhibit osteoclastogenesis and subsequent bone resorption through direct effects on osteocyte and MSC expression of RANKL, it is known that mechanical effects on bone remodeling also involve regulation of MSC differentiation toward osteogenesis (93, 114, 115) facilitated by Wnt/β–Catenin signaling and uncommitted precursors. This is, in part, due to the plasticity of stem cells to differentiate specifically toward one mesenchymal lineage over another as dictated by environmental cues, such as exogenous mechanical stimuli (93), local substrate rigidity (113, 116, 117), and regional cytokine signaling gradients (11, 118). These factors drive MSC and other resident marrow cells toward fulfilling their role in musculoskeletal homeostasis by promoting formation of bone and other critical tissues that support skeletal health in lieu of engaging pathways conducive to adipogenesis. Exercise not only encourages MSC proliferation but downstream lineages are also influenced as well: lipid droplets and adipocyte cell diameters are reduced while driving osteogenic potential through upregulated alkaline phosphatase activity (119). *In vivo* studies show decreased adipocytes and increased pre-osteoblasts in the marrow of running rats (38) and climbing mice (120).

Rodent studies highlight increased bone formation rates in response to exercise and mechanical signals *via* dynamic histomorphometry (104), including running exercise (121–124). These responses persist through incorporation of non-exercise mechanical loading interventions [low-magnitude mechanical signals (LMMS)], which have been demonstrated to increase bone formation rates in loaded tibiae of mice (125, 126). In consideration of the phenotypic differences in lineage subtypes across niches, the bone marrow-derived MSC has recently been suggested to have unique, focal-specific properties (127). Therefore, it is important to weigh the contribution of precursor cells and other progenitors in the presence of mature adipocytes or the marrow, when considering the effect mechanical stimuli may have on their interaction with the surrounding milieu.

### Low-Magnitude Mechanical Signals Effect on Bone and Marrow Adiposity

While physical activity presents an ideal strategy to introduce exogenous low-frequency, high-magnitude mechanical cues to musculoskeletal tissue, this approach is impractical for those patients with compromised bone microarchitecture (i.e., osteoporosis, osteopenia) (105, 109, 128–130) or muscle instability (131–133), populations that could benefit the most from their effects. Alternatively, mechanical signals delivered to the skeleton in the form of high-frequency, LMMS (fast-twitch muscles controlling balance and posture) can be introduced outside of the context of physical activity (134). For instance, low strain (<100 μs) displacements contribute more toward maintaining musculoskeletal health than higher magnitude strains and can be delivered whole-body using platforms to oscillate in the high-frequency domain (20–100 Hz), while maintaining a low-magnitude (i.e., sub-gravitational) acceleration. In doing so, these platforms partially reintroduce the spectral content of muscle contraction (105, 135), thereby exerting a beneficial quotient of exercise without risking fracture to bone resulting from extreme loads prevalent in exercise. Moreover, when separated into multiple administrations, the effects of both exercise and LMMS are amplified (95), encouraging enhancement in the responsiveness of MSC. Importantly, in humans, the non-pharmacologic therapy LMMS prevent bone loss due to Crohn’s disease (136), in children recovering from various cancers (137), and in other disabling conditions where bone losses are apparent (138, 139).

## Exercise and the Browning of Marrow Adipose Tissue

Upon initiation of exercise, there is an increase in uptake and oxidation of lipids in skeletal muscle (140). When exercise intensity increases, fuel selection shifts toward an increase in carbohydrate and decrease in fat utilization. In contrast, endurance training is associated with a shift toward an enhanced lipid utilization (140). BAT, initially observed in hibernating mammals and human infants, dissipates energy in the form of heat through non-shivering thermogenesis (141). Inducible brown fat depots – beige fat – have been discovered within the white adipose tissue of adult humans (142). On exposure to cold or β-adrenergic stimulation, these beige/brite fat cells express high levels of mitochondrial uncoupling protein UCP1 and fat globules become multilocular (143), characteristics of the brown fat phenotype. Irisin, a muscle-derived hormone induced by exercise, also activates UCP1 expression and browning of white adipose tissue (33): coactivator PPAR-γ coactivator-1 α (PGC1-α) stimulates irisin, and transgenic mice overexpressing PGC1-α exhibit increased energy expenditure despite no changes in food intake or activity (33). Overall, there is evidence that fat depots can alter phenotype to serve functional demands. Since exercise browns white adipose depots (33), it is conceivable that exercise might result in analogous browning of MAT. Indeed, running exercise increased UCP1 in bone mRNA (48). UCP1 is localized in the mitochondrial inner membrane of mammalian BAT and is, therefore, a specific marker for BAT (144). This increase in UCP1 with exercise may indicate a brown phenotype within MAT adipocytes; however, this requires further confirmation. Interestingly, exercise-induced increases in UCP1 expression may correlate with increases in irisin (33), although irisin’s role in exercise physiology remains unclear (145). Finally, recent work suggests that irisin may have direct effects on bone in addition to its known effect on adipocytes, and thus irisin’s role in skeletal health remains an area of active investigation (146–148).

## Conclusion

Marrow adipose tissue, housed within bone and interspersed with hematopoietic elements, remains a poorly understood fat depot, likely due to its anatomic location, rendering it inaccessible and thus challenging to quantify. Clinicians are particularly interested in the physiology of this fat depot due to its association with low bone density states and pharmacologic agents that increase fracture risk. As more robust volumetric imaging and quantification tools emerge [e.g., osmium-μCT Ref. (13, 48)], precise determinations can be made regarding MAT physiology and relationship to bone health. Interestingly, these methodologies have pointed to an increase in MAT in the setting of high-fat feeding (13), without an impact on bone quantity. Additionally, we are now able to visualize the effect of pharmacologic PPARγ activation with rosiglitazone on bone and to appreciate the significant encroachment of marrow fat (Figure 2) as well as to quantify these dramatic findings. Interestingly, both regimented exercise and LMMS serve to counter the effects of obesity and potent pharmacologic agents on bone remodeling. Further, in evaluating the response of MAT to mechanical stimuli, we highlight a positive effect toward normalizing bone parameters and, in doing so, constraining expansion of MAT across the marrow. It is possible that exercise serves to “brown” MAT as indicated in Ref. (48); however, further work is needed to establish the metabolic purpose of MAT in the setting of exercise. It remains unclear whether exercise-induced bone formation biases MSCs away from adipogenesis in order to recruit osteoblasts or whether alternative mechanisms are involved. *In vitro*, MSCs are highly responsive to mechanical signals during differentiation; indeed, mechanical loading slows adipogenesis (38, 92, 93, 149–151). Conversely, MSCs under microgravity conditions decrease osteogenic differentiation in favor of fat formation, an event accompanied by elevated nuclear expression of PPARγ (152). Thus, in the setting of mechanical input or exercise, bone formation is increased and marrow fat is suppressed, highlighting a likely mechanistic relationship between MAT and bone in the setting of mechanical stimulation. Other pathways are likely involved in the exercise regulation of MAT: lipogenesis, lipid uptake, skeletal anabolism, regulation of hematopoiesis in the bone marrow, and regulation of adipokines and cytokines. The elucidation of these pathways and their role in MAT/bone regulation in the setting of exercise remains an area of active investigation.

## Author Contributions

MS and GP wrote this review together as a team. Each author contributed approximately 50% to the writing of this review.

## Conflict of Interest Statement

The authors declare that the research was conducted in the absence of any commercial or financial relationships that could be construed as a potential conflict of interest.
